# Estimating the impact of improved management of haemophilia a on clinical outcomes and healthcare utilisation and costs

**DOI:** 10.1186/s13104-023-06552-3

**Published:** 2023-11-10

**Authors:** Ravichandran Chandrasekaran, Mauro Dávoli, Zulaiha Muda, Uendy Pérez-Lozano, Naouel Salhi, Nakul Saxena, Ming-Ching Shen, HyeRyoung Haylee Song, Darintr Sosothikul, Veronica Soledad Soto-Arellano, Igor Solev

**Affiliations:** 1 Department of Paediatric Hematology & Oncology, Institute of Child Health and Hospital for Children, Madras Medical College, Chennai, Tamil Nadu India; 2 Rosario Hemophilia Fundación, Rosario-Santa Fé, Argentina; 3 Paediatric Department, Hospital Tunku Azizah Women Children Hospital, Kuala Lumpur, Malaysia; 4 Hematology Service, Unidad Médica de Alta Especialidad “Manuel Avila Camacho”, Instituto Mexicano del Seguro Social, Puebla, Mexico; 5 Department of Hematology, University Hospital of Constantine, Constantine, Algeria; 6 Department of Internal Medicine, Changhua Christian Hospital, Changhua, Taiwan; 7 Takeda Pharmaceuticals International AG Singapore Branch, Singapore, Singapore; 8 Integrative and Innovative Hematology/Oncology Research Unit, Division of Hematology/Oncology, Department of Paediatrics, Faculty of Medicine, Chulalongkorn University, Bangkok, Thailand; 9 Department of Hematology, Hospital Roberto del Río, Santiago, Chile

**Keywords:** Bleeds, Haemophilia a, Cost of illness, Healthcare resource use, Costs

## Abstract

**Objective:**

Haemophilia A (HA) is associated with high clinical and healthcare burden. We developed an Excel-based model comparing current practice to improved management in severe HA patients currently managed on demand (OD). Outcomes included short- and long-term bleed events. Expected annual bleeds were estimated based on locally-derived OD annualised bleed rate (ABR), adjusted by relative prophylaxis-related ABRs (published literature). The objective of our study was to explore the impact of improving HA prophylaxis in target countries with limited published data (Algeria, Argentina, Chile, India, Malaysia, Mexico, Taiwan and Thailand). Bleed-related healthcare resource use (HCRU) and costs were estimated as a function of bleed type, with inputs obtained from local expert estimates. Clotting factor concentrates (CFC) consumption related to treatment and prophylaxis was estimated based on locally relevant dosing. CFC costs were not included.

**Results:**

When 20% of OD patients were switched to prophylaxis, projected reduction in bleeds was estimated between 3% (Taiwan) through 14% (Algeria and India); projected reductions in hospitalisations ranged from 3% (Taiwan) through 15% (India). Projected HCRU-related annual cost savings were estimated at USD 0.45 m (Algeria), 0.77 m (Argentina), 0.28 m (Chile), 0.13 m (India), 0.29 m (Malaysia), 2.79 m (Mexico), 0.15 m (Taiwan) and 0.78 m (Thailand). Net change in annual CFC consumption ranged from a 0.05% reduction (Thailand) to an overall 5.4% increase (Algeria). Our model provides a flexible framework to estimate the clinical and cost offsets of improved prophylaxis. Modest increase in CFC consumption may be an acceptable offset for improvements in health and healthcare capacity in resource constrained economies.

**Supplementary Information:**

The online version contains supplementary material available at 10.1186/s13104-023-06552-3.

## Introduction

Haemophilia A (HA) is a debilitating disorder associated with high clinical and healthcare burden, disproportionately impacting lower-middle income countries (LMICs) [1, 2]. Long-term prophylaxis with clotting factor concentrates (CFC) improves outcomes by reducing bleeding events and improving long-term musculoskeletal and quality of life outcomes [2]. However, the management of HA varies substantially across LMIC and higher income countries, and the relative reductions in healthcare resource utilization (HCRU) achievable through improved management of HA have not been well explored due to limited country-level data.

The goal of the World Federation of Hemophilia (WFH) is to reach a target annualised bleed rate (ABR) of zero through improvement in haemophilia management [2, 3]. Latest reports from the WFH World Bleeding Disorders Registry (WBDR) indicate that real-world practice still has a long way to go to meet these goals, due to continued geographical disparities in country-reported ABRs and limited use of prophylaxis in both paediatric and adult populations [3]. The WFH 2020 management guidelines indicate high dose prophylaxis as the gold standard of management for patients with severe haemophilia [3]. The guidelines emphasise that on demand (OD) management does not meet patient goals of full and safe management of bleeding events, but acknowledge that in resource-constrained environments, lower dose prophylaxis regimens can constitute a pragmatic first step toward optimising HA management [3]. Despite recommendations, prophylaxis is perceived as expensive, with CFCs historically constituting up to 80% of the direct cost of management [4, 5]. Understanding the potential offsets of improved HA management in the context of inpatient and outpatient activity could help facilitate a move toward improving HA management in countries with limited resources or cost constraints.

This study explored the impact of moving toward improved management of HA across a range of North African, Latin American and South-East Asian countries, where accessible data on the current burden of HA is limited and a range of management strategies are employed. Quantification of the outcomes of current management strategies allows for a clear picture of potential reductions in bleed-related HCRU with a shift to improvement. The primary objective of the research was therefore to estimate the current clinical and HCRU-related cost burden of HA and explore the potential clinical and cost impact of a pragmatic move toward improved management for patients currently managed OD.

## Materials and methods

### Overview

A prevalence-based annual burden of illness (BOI) model was developed to better understand the clinical and economic landscape of HA in target countries where published data are scarce. The model was built as a flexible platform to explore current and future HA management scenarios across multiple geographies. The research reported in this paper includes 8 countries: Algeria, Argentina, Chile, India^1^, Malaysia, Mexico, Taiwan and Thailand. Costs were estimated from the perspective of the national healthcare payer with indirect costs explored in additional analysis. Costs were estimated in local currency and converted to 2020 USD for comparability of outcomes.

### Model development and structure

An Excel-based model was used to estimate the current clinical outcomes and costs of HA. All patients diagnosed with HA were included in the model. Key characteristics of the countries and cohorts are reported in Table 1. Outcomes included short- and long-term bleed events based on annual bleed rates (ABRs) (classified as joint bleed (JB), other major bleed (OMB), non-major bleed (NMB) and permanent joint damage). Bleed-related healthcare resource use (HCRU) and costs were estimated as a function of bleed type. The clinical and cost outcomes associated with current management were compared against a hypothetical counterfactual where management of patients with HA was moved toward improved prophylaxis (and therefore reduced ABR). The model structure comprised a simple calculation matrix where bleed events, HCRU and costs were tallied across the cohorts according to the distribution of management strategies (see Table 1). The difference in outcomes and costs between the two cohorts allowed the estimation of the potential health and cost impact of a move toward improved management of HA in severe HA patients currently managed OD.

**Table 1:** Current Haemophilia A management characteristics

Characteristics		Algeria	Argentina	Chile	India	Malaysia	Mexico	Taiwan	Thailand
**Income category** [6]		LMIC	UMIC	HIC	LMIC	UMIC	UMIC	HIC	UMIC
**PWHA, n** [2, 7]		1,911	3,069	1,566	2,000^†^	950	4,814	992	1,557
**Patients with severe HA, %**		90%	69%	30%	82%	59%	68%	72%	59%
**Children, %**		37%	33%	30%	30%	43%	38%	33%	75%
**Current Practice**	**OD**	62%	55%	74%	91%	50%	28%	28%	78%
	**VLD SHL Px**	0%	0%	0%	2%	0%	0%	0%	8%
	**LD SHL Px**	0%	0%	5%	7%	23%	0%	0%	14%
	**ID SHL Px**	38%	1%	21%	0%	19%	15%	15%	0%
	**HD SHL Px**	0%	38%	0%	0%	5%	0%	0%	0%
	**Pers. SHL Px**	0%	6%	0%	0%	0%	0%	0%	0%
	**EHL Px**	0%	0%	0%	0%	4%	57%	57%	0%
**Home-Based Px (%)**		65%	90%	90%	0%	86%	90%	85%	90%
**Compliance (%)**		100%	65%	75%	50%	80%	74%	95%	80%
**OD annual bleeds** ^**‡**^		36.0	17.5	25.0	17.5	12.0	33.0	31.9	22.0
**Patients switched from OD (n)** ^††^		209	157	25	290	21	78	4	130

Notes: ^†^Estimated population of patients with haemophilia A in India (Tamil Nadu)^; ‡^The estimated annual bleed rate of an adult patient with severe haemophilia A managed without prophylaxis (Px ABRs are estimated as a function of the reported OD ABR); ^††^The switch population comprised 20% of the severe HA patients currently managed via an OD regimen

**Abbreviations**: EHL: extended half-life; HA: haemophilia A; HIC: high income country; ID: intermediate-dose; LD: low-dose; LMIC: low-middle income country; Pers.: personalised; Px: prophylaxis; PWHA: patients with haemophilia A; SHL: standard-half life; UMIC: upper-middle income country; VLD: very low-dose

### Local clinician expert interviews

Expert interviews (on average, three haematologists per country) were conducted to ensure local applicability of the country-specific models. Structured searches designed to populate the core model parameters were conducted across all target country settings, but limited candidate data were retrieved for input to the model. A detailed discussion guide was developed to fill data input gaps and inform core local data on current management and plausible shifts in treatment improvement. A sample of haematologists actively involved with the management of HA in local haemophilia treatment centres (HTC) was interviewed in each country to validate the patient pathways and data gaps. Data inputs derived from expert interviews included country-specific OD ABRs, current management of HA (including intensity of dosing and setting for the delivery of prophylaxis), epidemiological breakdown of bleeds, HCRU associated with the management of bleeds and the unit costs of health service delivery (where local published tariffs were unavailable). See supplementary files, Table A1 for HCRU details.

### Estimation of annual bleeds

There are limited studies conducted outside of Europe and America to usefully inform locally-relevant estimates of underlying ABR [8]. Expected annual bleeds associated with current practice were therefore estimated based on locally-defined OD ABRs with an adjustment made for the proportion of patients currently receiving prophylaxis regimens (Table 1). There was wide variety in the estimate of the expected OD ABR that were provided by the interviewed clinicians across the included countries. These differences reflect the varying standards of care across the countries. Prophylaxis-related ABR adjustments were estimated relative to OD ABRs based on available data from long-term clinical trials (see supplementary Table A2 for calculation inputs). This approach was taken to enable estimates of any composite of local practice without requiring local clinicians to estimate ABRs for each individual management strategy. A recent international trial reporting comparative long-term real-world outcomes in patients managed either OD or with standard half-life (SHL) prophylaxis was used as the benchmark for the ABR calculations [9]. Expected ABRs for extended half-life (EHL) prophylaxis were estimated based on recent trials [10, 11]. Expected ABRs for lower intensity regimens were estimated based on a recently reported simulation exercise [12]. The calculation framework allowed estimation of the expected annual bleeds for any composite of locally relevant management practice.

### Estimation of bleed-related HCRU and costs

Initial literature searches conducted to inform data gaps in model inputs indicated limited open-source HCRU and cost data across the 8 countries. Candidate data generated from clinician expert interviews were used to inform expected management of bleed events. HCRU estimates were provided as a function of type of bleed and probability of healthcare professional (HCP) contact. Resource components included nurse or clinic contacts, outpatient attendances, hospitalisations (and estimated length of stay), diagnostics (MRI and CT scans), surgery for long-term complications (where country practice dictated this) and the expected costs associated with post-surgery rehabilitation. The cost of events was then estimated by combining local healthcare resource use with the relevant local unit cost. In the absence of open-source cost tariffs, costs were provided by the local clinician experts. Note that when data were synthesised, extreme outliers of response were excluded and a simply calculated mean value was used in the country models. HCRU and unit cost inputs are detailed in Table A1 in the supplementary files.

### Estimation of CFC consumption and prophylaxis clinic visits

Total CFC consumption related to both treatment and prophylaxis of bleeding events was estimated based on published dose instructions taking account of type of bleed, IU per kg dose, and frequency and duration of delivery [1, 13]. Not all bleeds lead to CFC consumption; in this exploratory analysis, the likelihood of receiving CFC was set to 75% for NMB, 90% for JB and 100% for OMB. These assumptions and calculations were applied consistently across all countries. Compliance was estimated based on expert feedback and used to down-adjust total prophylaxis-related CFC consumption (but did not impact estimated ABRs). The additional clinic costs of prophylaxis delivery were incorporated for those patients managed in a clinic setting. No additional healthcare payer costs were applied for home-based delivery of prophylaxis. CFC costs were not included but the expected change to per capita CFC consumption is estimated and clearly reported. CFC costs were excluded from the analysis due to insufficient information on potential procurement arrangements, preventing robust estimation of a standard unit cost applicable at the country level. Estimate of the per capita consumption allows for a transferable and generalisable metric that can be interpreted and explored at a local level to facilitate local decision making.

### Incorporation of long-term and indirect costs

Additional analysis explored the annualised impact of the longer-term complications of HA and incorporated indirect costs associated with time off work. A proportion of HA patients will experience the impact of target joint bleeds and potential long-term disability [3]. The model also assumed that patients experiencing a bleed will have time off work or school. Inputs were based on expert-derived estimates relating to duration of bleed and/or length of stay in hospital. Time off work was costed by applying the median daily wage to all days lost from work for each respective country, taking a simple human capital approach to the valuation of lost productivity. The impact on carer time for minors missing school was not included. Double counting was avoided by ensuring that the total sum of days lost (either from school or work) was no longer than the stated duration of bleed. Out of pocket costs (e.g. transport) were not included.

### Switch to improved management

The model was built as a flexible platform to explore different scenarios of prophylaxis treatment strategies; the majority of target countries were already moving toward improved HA management in the form of higher dose SHL prophylaxis or EHL prophylaxis. In these first exploratory analyses, we considered a switch, where 20% of severe HA patients that were still managed OD were switched to intermediate dose prophylaxis (ID Px) (7 target countries) or to EHL prophylaxis (only in Taiwan). Estimates of annual bleeds with improved prophylaxis were calculated by adjusting the estimates of current bleeds (see above) to reflect the changed management profile. Bleed-related events, bleed-related HCRU and associated direct and indirect costs were estimated and compared against the current burden (no change to management) to quantify the clinical and economic benefits of management improvement. The numbers of patients managed OD differed across countries and this influenced the numbers of patients who switched treatment (Table 1). However, the relative reduction in bleed rates was applied consistently across all country analyses.

### Treatment of uncertainty

Owing to the lack of published data in target countries, there were a large number of expert-provided input in the models and therefore a high degree of input uncertainty. Extensive one-way sensitivity analyses (OWSA) were conducted to help understand key model drivers and focus future research. Probabilistic sensitivity analysis was not conducted for these exploratory analyses as the evidence generated within the research did not provide enough granularity to define plausible distributions and defendable limits around the candidate set of data inputs. This is in line with the primary focus of exploratory analyses where the aim is to explore broader trends rather than produce precise probabilistic estimates.

## Results

This study reported an estimate of the annual burden of HA for each target country alongside preliminary estimates of the country-specific impact of locally plausible changes to current HA management.

### Current clinical burden

Current practice and management of HA differed substantially across countries (Table 2). Total per country bleeds were estimated at between 3,900 (Malaysia) and 45,100 (Mexico), with per patient annual bleed rates varying substantially between an estimated 4 per patient in Malaysia and 21 per patient in Algeria. HCRU differed due to both number of bleed events and reported country practice in the management of bleeds. The annual burden of clinic and outpatient attendance for treatment of bleed-related events was estimated at between 3,600 (Malaysia) and 120,500 (Mexico) attendances. The annual number of inpatient episodes due to bleed-related events (including acute and longer term management) was estimated at between 478 (Argentina) and 24,300 (Mexico). Across all countries, most patients receive home-based prophylaxis however, current annual CFC-related attendances for in-clinic prophylaxis were estimated at between 2,900 visits (Thailand) and 43,300 visits (Mexico).

**Table 2:** Estimated current annual clinical and economic burden of HA

Total Outcomes		Algeria		Argentina		Chile		India		Malaysia		Mexico		Taiwan		Thailand	
		Total	Per PWHA	Total	Per PWHA	Total	Per PWHA	Total	Per PWHA	Total	Per PWHA	Total	Per PWHA	Total	Per PWHA	Total	Per PWHA
**Bleeds (n)**		40,020	20.94	20,853	6.80	5,934	3.79	26,661	13.33	3,893	4.10	45,119	9.39	3,957	3.99	16,241	10.43
**IP Episodes (n)**		890	0.47585	478	0.16	5,370	3.43	3,683	12.69	1,092	1.15	24,293	5.06	605	0.61	1,581	1.87
**Tx Visits (n)**		11,443	5.99	43,792	14.28	5,996	3.83	6,246	3.12	3,644	3.84	120,504	25.08	5,346	5.39	4,016	2.58
**Px Visits (n)**		32,592	17.05	21,321	6.95	5,386	3.44	19,138	9.57	8,479	8.93	43,351	9.02	13,855	11.37	2,906	1.87
**Target Joints (n)**		17,953	9.39	8,851	2.89	1,580	1.01	15,901	7.95	2,029	2.14	11,788	2.45	1,259	1.27	7,423	4.77
**Surgeries (n)**		1,585	0.83	1,726	0.56	783	0.50	463	0.23	447	0.50	597	0.12	50	0.05	99	0.06
**Deaths**		9	0.00	15	0.00	1	0.00	9	0.00	1	0.00	72	0.00	2	0.00	8	0.00
**Missed Days**		186,647	95	101,281	33.03	82,198	52.49	60,326	30.16	40,312	42.44	252,932	52.64	9,409	7.43	109,152	63.73
**CFC Consumption (IU)**		320.04 M	167,475	371.39 M	121,084	92.91 M	59,327	49.13 M	24,563	57.20 M	60,206	806.50 M	167,851	193.90 M	195,467	94.32 M	60,579
**Costs (USD)**	**HCRU**	2.35 M	1,232	6.16 M	2,008	0.52 M	335	1.01 M	533	3.51 M	3,690	60.46 M	12,583	4.22 M	4,256	9.45 M	6,069
	**Indirect**	1.84 M	964	1.37 M	448	2.64 M	1,684	0.27 M	135	1.22 M	1,286	3.83 M	797	0.66 M	667	1.02 M	656
	**Total**	4.20 M	2,196	7.53 M	2,455	3.16 M	2,019	1.33 M	667	4.73 M	4,976	64.29 M	13,380	4.88 M	4,923	10.47 M	6,725

**Abbreviations**: CFC: clotting factor concentrates; HA: haemophilia A; HCRU: healthcare resource use; IP: Inpatient (hospitalisations); IU: international units; PWHA: patient with haemophilia A; Px: prophylaxis; Tx: treatment

### Baseline economic burden

HCRU costs were estimated at USD 2.35 m (Algeria), 6.16 m (Argentina). 0.52 m (Chile), 1.01 m (India), 3.51 m (Malaysia), 60.46 m (Mexico), 4.22 m (Taiwan) and 9.45 m (Thailand), equating to a per patient annual cost (excluding CFC consumption) of USD 1,232 (Algeria), 2,008 (Argentina), 335 (Chile), 533 (India), 3,690 (Malaysia), 12,584 (Mexico), 4,256 (Taiwan) and 6,069 (Thailand).

Time off work and school were estimated for each country, and time off work was used to estimate country-specific indirect costs. When indirect costs were included, costs were estimated at USD 4.20 m (Algeria), 7.53 m (Argentina), 3.16 m (Chile), 1.33 m (India), 4.73 m (Malaysia), 64.29 m (Mexico), 4.88 m (Taiwan) and 10.47 m (Thailand). Note that caregiver indirect costs were not included in the analysis base case.

### Impact of a move toward improved management

**Fig. 1:**
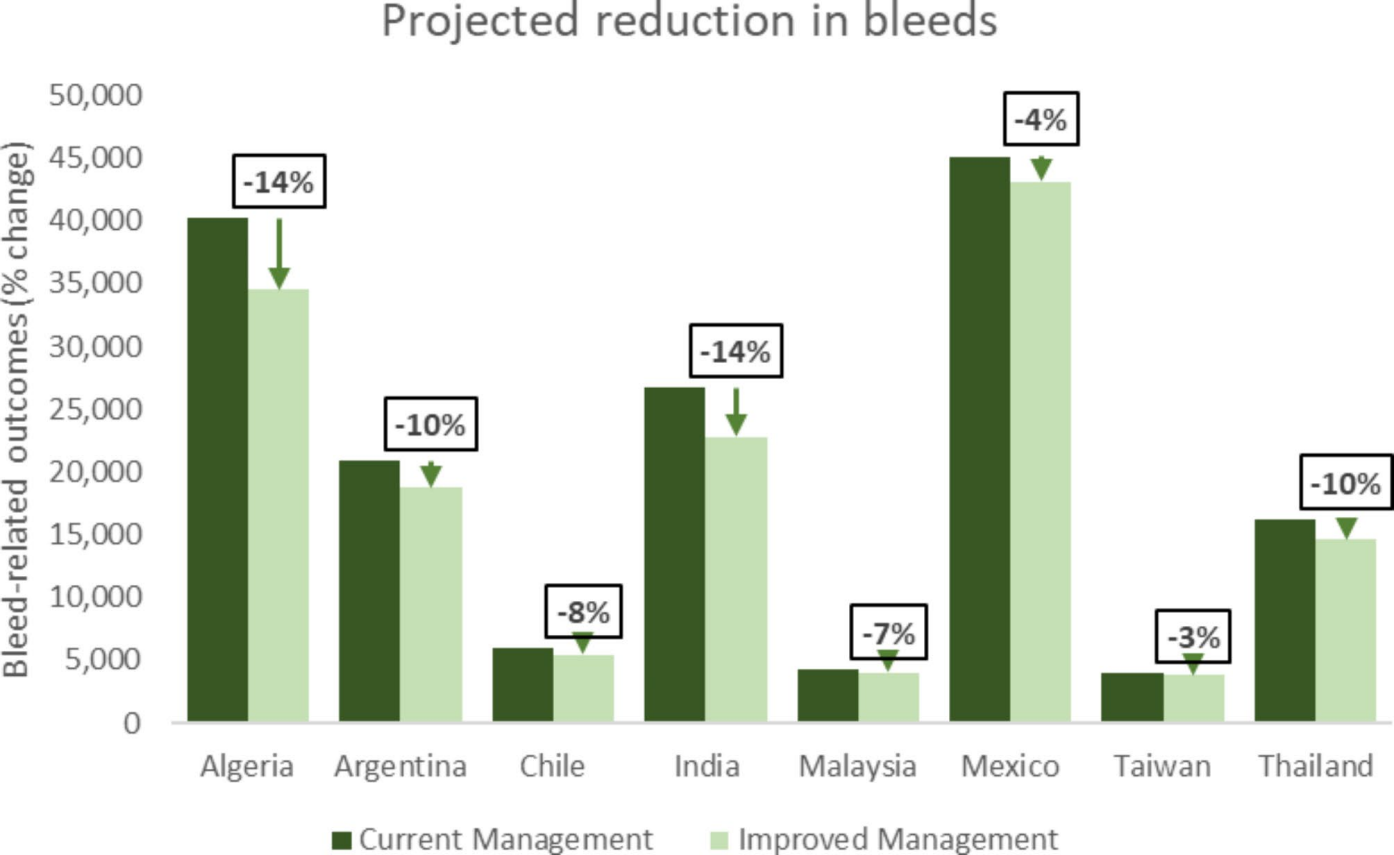
Projected impact on bleeds following change to HA management

**Fig. 2:**
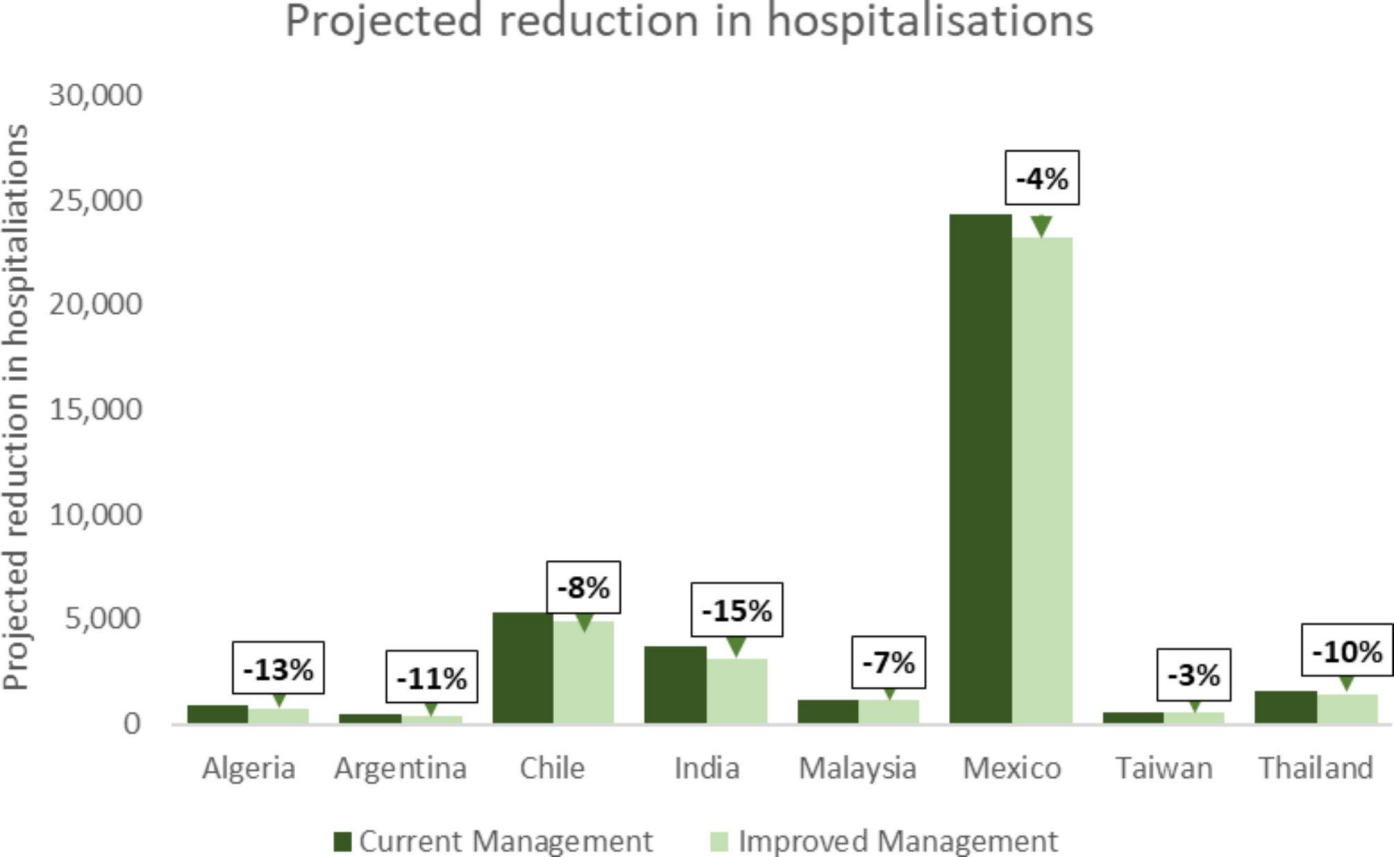
Projected impact on hospitalisations following change to HA management

Substantial reductions in bleed events were projected through the modelled switch, with an estimated range of between 131 (Taiwan) and 5,485 (Algeria) bleeds avoided per year. HCRU impact differed due to both number of bleed events and reported country practice in the management of bleeds, however, substantial reductions in bleed-related HCRU were projected across all country settings (Table 3). Projected reduction in annual bleeds ranged from 3% (Taiwan) through 14% (Algeria and India) (Fig. 1) and projected reductions in hospitalisations ranged from 3% (Taiwan) through 15% (India) (Fig. 2).

**Table 3:** Impact of change in HA management on clinical outcomes and annual costs

Impact on outcomes with improvements in HA management		Algeria		Argentina		Chile		India		Malaysia		Mexico		Taiwan		Thailand		
		Total	Per switcher	Total	Per switcher	Total	Per switcher	Total	Per switcher	Total	Per switcher	Total	Per switcher	Total	Per switcher	Total	Per switcher	
**Bleeds (n)**		-5,485	-26.24	-2,169	-13.82	-475	-19.00	-3,856	-13.30	-214	-10.193	-1,984	-25.44	-131	-32.75	-1,656	-12.74	
**IP Episodes (n)**		-115	-0.55	-53	-0.34	-430	-17.20	-535	-1.84	-61	-2.903	-1,081	-13.86	-20	-5.00	-161	-1.24	
**Tx Visits (n)**		-1,554	-7.44	-4,649	-29.61	-470	-18.80	-906	-3.12	-203	-9.67	-5,321	-68.22	-176	-44.00	-410	-3.15	
**Px Visits (n)**		+ 9,459	45.26	+ 2,454	15.63	+ 327	13.08	+ 30,149	103.96	+ 443	+ 21.10	+ 1,013	12.99	+ 79	19.75	+ 1,694	13.03	
**Target Joints (n)**		-2,554	-12.22	-959	-6.11	-132	-5.28	-2,316	-7.99	-130	-6.19	-538	-6.90	-41	-10.25	-764	-5.88	
**Surgeries (n)**		-225	-1.08	-187	-1.19	-67	-2.68	-68	-0.23	-29	-1.38	-28	-0.36	-2	-0.50	-10	-0.08	
**Deaths**		-1	0.00	-2	-0.01	-0	0.00	-1	0.00	-0	0.00	-4	-0.05	-0	0.00	-1	-0.01	
**Missed Days**		-25,648	-123	-10,800	-69	-6,797	-272	-8,744	-30	-2,4888	-118	-11,568	-148	-312	-78	-10,126	-78	
**CFC Consumption (IU)**		+ 17.12 M	+ 81,897	+ 3.54 M	+ 22,568	-1.46 M	-58,461	+ 6.60 M	+ 22,742	-1.69 M	-80,60	+ 9.71 M	+ 124,481	+ 0.05 M	+ 13,673	+ 0.05 M	+ 364	
**Costs (USD)**	**HCRU**	-0.19 M	-888	-0.59	-3,753	-0.03	-1,087	-0.09 M	-316	-0.21 M	-9,974	2.59	-33,170	0.13	-33,334	0.68	-5,213	
	**Indirect**	-0.27 M	-1,273	-0.17	-1,088	-0.25	-10,082	-0.04 M	-139	-0.08 M	-3,823	0.21	-2,636	0.02	-5,415	0.11	-818	
	**TOTAL**	-0.45 M	-2,160	-0.76	-4,841	-0.28	-11,169	-0.13 M	-455	-0.29 M	-13,797	2.79	-35,805	0.15	-38,748	0.78	-6,031	

**Notes** Cost savings incorporate avoidance of HCRU costs associated with the long-term management of bleeds

**Abbreviations**: CFC: clotting factor concentrates; HCRU: healthcare resource use; IP: Inpatient (hospitalisations); IU: international units; PWHA: patient with haemophilia A; Px: prophylaxis; Tx: treatment.

**Fig. 3:**
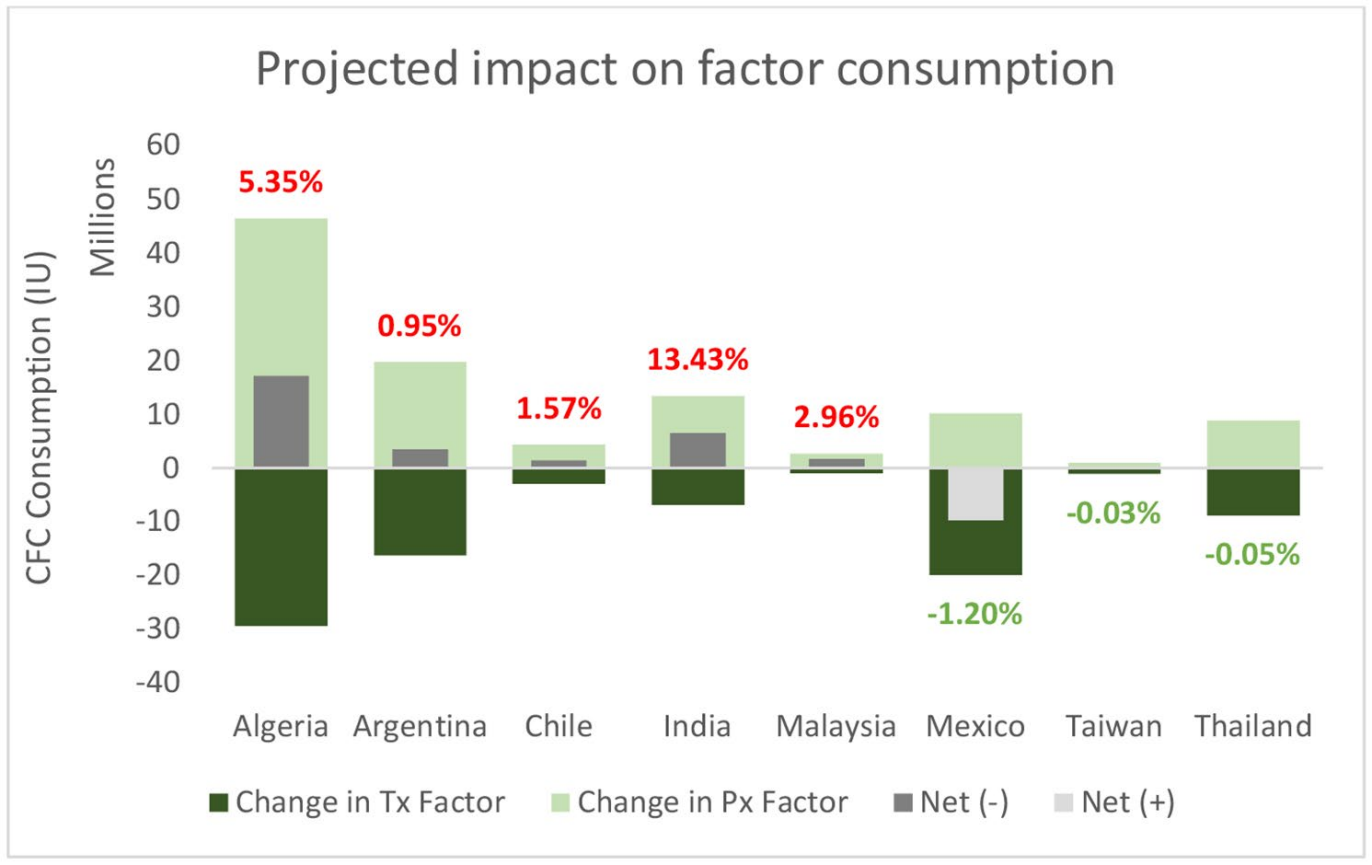
Projected impact on CFC consumption following change to HA management

Increases in uptake of CFC through increased prophylaxis were in part offset by decreases in CFC consumption related to treatment of bleeds, with the net change in CFC consumption ranging from a 0.05% reduction (Thailand) through to a 5.35% increase (Algeria) (Fig. 3).

Overall direct health system savings through reduction in bleed-related HCRU were estimated at USD 0.19 m (Algeria), 0.59 m (Argentina), 0.03 m (Chile), 0.09 m (India), 0.21 m (Malaysia), 2.59 m (Mexico), 0.13 m (Taiwan) and 0.68 m (Thailand). In all countries, the cost of additional prophylaxis-related clinic visits was offset by bleed-related HCRU savings. When including indirect costs, potential total annual cost saving estimates rose to USD 0.45 m (Algeria), 0.77 m (Argentina), 0.28 m (Chile), 0.13 m (India), 0.29 m (Malaysia), 2.79 m (Mexico), 0.15 m (Taiwan) and 0.78 m (Thailand). Note that caregiver lost productivity was not considered.

### Sensitivity analyses

OWSA indicated the key drivers of model outcome in each country analysis (Table 4). Common drivers of outcome were found across country markets, with baseline ABR and percentage of patients switching from OD management the top drivers of influence across all country analyses. In exploratory models such as this, findings from the OWSA can help focus additional research by identifying parameters that are currently assumption-led but substantially impact model outcomes.

**Table 4:** Key drivers of model outcomes from one way sensitivity analysis

Model input	Algeria	Argentina	Chile	India	Malaysia	Mexico	Taiwan	Thailand
**Baseline ABR**	✓	✓	✓	✓	✓	✓	✓	✓
**Switch (%)**	✓	✓	✓	✓	✓	✓	✓	✓
**Resource (JB)**	✓		✓			✓	✓	✓
**Population**	✓		✓		✓			
**Joint Bleed (%)**		✓		✓	✓			
**Cost of LTD**		✓		✓	✓			
**Inpatient daily cost**						✓	✓	✓
**Target Joint (%)**		✓		✓				
**Resource (OMB)**						✓	✓	
**Daily wage**	✓		✓					
**Home prophylaxis (%)**								✓

**Abbreviations**: ABR: annualized bleeding rates; JB: joint bleeds; LTD: long-term disability; OMB: other major bleeds; ✓ indicates the 5 parameters most influential on the estimation of total annual cost of care for HA.

**Abbreviations**: CFC: clotting factor concentrates; Px: prophylaxis; Tx: treatment

## Discussion

This was an exploratory model developed across multiple geographies with input from local experts. The model allowed exploration of a series of locally relevant scenarios, taking into account the current standard of care and locally relevant switch scenarios, including switch to low dose prophylaxis regimens. Based on current assumptions we found that optimisation of HA management, through switching a proportion of patients currently managed OD could result in substantial reduction in bleed events and bleed-related HCRU and cost.

The model estimated current annual bleeds per patient between 4 (Malaysia) and 21 (Algeria), with per patient annual HCRU cost burden estimated between USD 335 (Chile), and USD 6,069 (Thailand). Switching 20% of patients currently managed OD to an improved management schedule could lead to a substantial reduction in annual bleed events (up to 14%), overall reductions in bleed-related HCRU, including up to 15% reduction in HA-related hospitalisations, and a reduction in HCRU costs of between USD 0.03 m (Chile) and USD 2.59 m (Mexico). Our analysis indicates that these improvements could be achieved with either minimal increase or, in some settings, reductions in overall CFC consumption.

The profile of switch and OD ABR differed by country, with numbers of patients with severe HA currently managed OD and switching to an improved regimen ranging from 4 patients (Taiwan) to 290 patients (India). However, improving treatment management strategies resulted in substantial reductions in bleeds and bleed-related HCRU and further, costs in those countries with higher rates of continued use of OD management (even when additional clinic visits for delivery of prophylaxis were taken into account). In addition, the increase in prophylaxis-related CFC consumption can be weighed against reductions in service provision and reductions in treatment-related CFC consumption through reduced bleed events. The core strength of this analysis is in the development of a flexible framework that allows exploration of a compelling economic case for targeted improvement of HA management despite limited local data. The analysis account for insight into the impact that improvement in HA management may have based on country-level outpatient and inpatient capacity. In countries where health services are already maximized, modest increases in CFC consumption may be an acceptable offset for improvements in healthcare capacity.

The recent WBDR report demonstrates continued unmet need to meet WFH targets of a zero bleed HA population [5]. In addition, geographical disparities are clear with median ABRs ranging from 6 in low and lower-middle income countries (range 3–11) to 2 in high-income countries (range 2–6). The report also emphasised the current low uptake of prophylaxis, with only 18% of HA patients receiving prophylaxis in the previous year, rising to 28% in patients with severe HA [3]. The WFH acknowledged that in countries with healthcare constraints, whilst prophylaxis should remain the gold standard, less intense doses can be used to manage costs [1]. Our model allows exploration of lower intensity dosing alongside exploration of more evolved care management (EHL prophylaxis).

BOI studies allow for the quantification of disease-related outcomes at a population level and are often used to advocate for a change in current practice [14]. Exploratory analyses can provide a benchmark estimate of the current clinical and economic burden of HA management where published real-world evidence is limited. Analyses indicate the potential for a reduction in CFC consumption for management of bleeds and substantial freeing up of healthcare services through improvement of HA management from OD to improved HA management.

These analysis outputs could be considered a useful first-line option to assess the potential impact of an improvement of current HA management strategies. Exploratory analyses such as those reported here may be especially useful in developing countries where resource constraints could limit adoption of high-dose prophylaxis and available data on current burden of HA is limited. Additional co-ordinated research to better estimate comparative ABRs and bleed-related HCRU is recommended and would increase the robustness of analysis outputs.

In summary, this study showed that the current burden of HA remains high despite shifts towards optimised management. Modest improvements in HA management could still lead to substantial reductions in bleed events and marked HCRU cost savings, alongside considerable impact on health system capacity. The core strength of this analysis is in the development of a flexible framework that allows exploration of a compelling economic case for targeted improvement of HA management despite limited local data. These outputs could be considered a useful first step in assessing the potential impact of an improvement in HA management, especially in developing countries where resource constraints can limit the adoption of high-dose prophylaxis.

### Limitations

This study is exploratory. Inputs were a mix of secondary research and expert discussions that might not reflect experience in an individual HTC [2, 7, 15]. Availability of robust comparative data is limited in HA [16, 17] and the relative ABR estimates utilised here should be considered provisional. Clinical outcomes associated with bleeds may be underreported if patients do not present to HTCs and it is possible that HCRU was underestimated by basing it on experience at a limited number of specialist HTCs. Compliance impacts only CFC consumption in our analysis and does not link to outcome as the relationship between adherence and outcome remains unclear [18]. Further research is needed on CFC use in real-world settings so that our analyses can be updated with local CFC consumption data and nationally relevant estimates of procurement costs. Finally, our estimates of burden do not include the costs and health impact of undiagnosed HA and inhibitor development as these constructs were outside the scope of the current model.

### Electronic supplementary material

Below is the link to the electronic supplementary material.

Supplementary Material 1

Supplementary Material 2

## Data Availability

All data generated or analysed are included in this published article and its supplementary information files.

## Footnotes

1 Note that for India, we refer to one state only (Tamil Nadu).
