# Supplementary material for: Estimating the impact of improved management of haemophilia a on clinical outcomes and healthcare utilisation and costs
Source: BMC Res Notes. 2023 Nov 10;16:327. doi: 10.1186/s13104-023-06552-3 (PMC10638687; doi:10.1186/s13104-023-06552-3)
Supplement: Supplementary file 1 — Supplementary Material 1 [file 13104_2023_6552_MOESM1_ESM.docx]

# **Impact of improved management of haemophilia A on the clinical and economic burden of disease in selected countries**

# **DISCUSSION GUIDE**

**EPIDEMIOLOGY**

1. **To reflect clinical practice, we are interested in categorizing patient outcomes by specific age groups. We have identified standardized age categories but please confirm with us what age bands drive your treatment choices.**

|  | Age range | Yes | No | If “No”, please provide the age bands that drive your treatment choices |
| --- | --- | --- | --- | --- |
| Children | 0 – 18 years old |  |  |  |
| Adults | >18 years old and above |  |  |  |

1. **Approximately how many patients with HA are there in Malaysia, and how many new patients with haemophilia A were presented to you in the past 1 year?**

|  | Age range | Population Size | Incidence rate (2020) |
| --- | --- | --- | --- |
| Children |  |  |  |
| Adults |  |  |  |

1. **What is the average weight of your patients?**

|  | Age range | Average Weight |
| --- | --- | --- |
| Children |  |  |
| Adults |  |  |

1. **What is the proportion breakdown of patients with HA in terms of severity and age groups?**

|  | Age range | Mild (%) | Moderate (%) | Severe (%) |
| --- | --- | --- | --- | --- |
| Children |  |  |  |  |
| Adults |  |  |  |  |

1. **A serious complication of factor replacement therapy is the development of inhibitors. What proportion of patients on average develop inhibitors annually?**

|  | Age range | Mild (%) | Moderate (%) | Severe (%) |
| --- | --- | --- | --- | --- |
| Children |  |  |  |  |
| Adults |  |  |  |  |

**TREATMENT PATHWAY**

1. **Based on published literature, we have defined the following treatment categories according to the FVIII dosage and frequency for patients with haemophilia A (without inhibitors). Please confirm if this is reflective of the treatment regimens in your country.**

| Treatment | Treatment Dosing |
| --- | --- |
| Episodic/on-demand (low dose) | Dependent on the type of bleed |
| Episodic/on-demand (standard dose) | Dependent on the type of bleed |
| Prophylaxis (low dose) | 10-15 IU/kg, 2 times per week |
| Prophylaxis (intermediate dose) | 15-25 IU/kg, 2 or 3 times per week |
| Prophylaxis (standard dose) | 25-40 IU/kg, 3 times per week |
| Prophylaxis (personalized) |  |

1. **What proportion of children/ adult patients with haemophilia A (without inhibitors) receive each of the below-listed treatment regimens in each age category?**

| Treatment regimen breakdown | Children (%) | | | Adult (%) | | |
| --- | --- | --- | --- | --- | --- | --- |
|  | Mild | Moderate | Severe | Mild | Moderate | Severe |
| Episodic/on-demand (low dose) |  |  |  |  |  |  |
| Episodic/on-demand (standard dose) |  |  |  |  |  |  |
| Prophylaxis (low dose) |  |  |  |  |  |  |
| Prophylaxis (intermediate dose) |  |  |  |  |  |  |
| Prophylaxis (standard dose) |  |  |  |  |  |  |
| Prophylaxis (personalized) |  |  |  |  |  |  |

1. **What proportion of children/ adult patients with haemophilia A receive home-based treatment?**

| Home-based prophylaxis | Children (%) | | | Adult (%) | | |
| --- | --- | --- | --- | --- | --- | --- |
|  | Mild | Moderate | Severe | Mild | Moderate | Severe |
| Episodic/on-demand (low dose) |  |  |  |  |  |  |
| Episodic/on-demand (standard dose) |  |  |  |  |  |  |
| Prophylaxis (low dose) |  |  |  |  |  |  |
| Prophylaxis (intermediate dose) |  |  |  |  |  |  |
| Prophylaxis (standard dose) |  |  |  |  |  |  |
| Prophylaxis (personalized) |  |  |  |  |  |  |

1. **How many patients do you expect to see switching to prophylaxis from episodic/ on demand treatment or low-dose prophylaxis treatment in the next few years?**

| Treatment switching | Children (%) | | | Adult (%) | | |
| --- | --- | --- | --- | --- | --- | --- |
|  | Mild | Moderate | Severe | Mild | Moderate | Severe |
| Episodic/on-demand (low dose) |  |  |  |  |  |  |
| Episodic/on-demand (standard dose) |  |  |  |  |  |  |
| Prophylaxis (low dose) |  |  |  |  |  |  |
| Prophylaxis (intermediate dose) |  |  |  |  |  |  |
| Prophylaxis (standard dose) |  |  |  |  |  |  |
| Prophylaxis (personalized) |  |  |  |  |  |  |

1. **What proportion of patients is on the respective factor replacement therapy?**

**Please feel free to specify any other treatment if your experience indicates differently.**

|  | Cryoprecipitate | Plasma-derived FVIII | Recombinant FVIII (Standard half-life) | Recombinant FVIII (Extended half-life) |
| --- | --- | --- | --- | --- |
| Children |  |  |  |  |
| Adults |  |  |  |  |

**TREATMENT PATHWAY**

1. **On average, how many bleeding events do HA patients experience in a year i.e., annual bleeding rate?**

| Annual Bleed Rates | Children (%) | | | Adult (%) | | |
| --- | --- | --- | --- | --- | --- | --- |
|  | Mild | Moderate | Severe | Mild | Moderate | Severe |
| Episodic/on-demand (low dose) |  |  |  |  |  |  |
| Episodic/on-demand (standard dose) |  |  |  |  |  |  |
| Prophylaxis (low dose) |  |  |  |  |  |  |
| Prophylaxis (intermediate dose) |  |  |  |  |  |  |
| Prophylaxis (standard dose) |  |  |  |  |  |  |
| Prophylaxis (personalized) |  |  |  |  |  |  |

1. **Given a bleed, what is the risk of different bleed types e.g., bleeds occurring in the major joints (ankles, knees, elbows, shoulders, wrists & hips) or other types of bleeding events?**

|  |  | Children (%) | Adult (%) |
| --- | --- | --- | --- |
| Bleeding events | What proportion of all bleeds are joint bleeds? |  |  |
|  | What proportion of all bleeds are major bleeds other than joint bleeds? |  |  |
|  | What proportion of joint bleeds are target joints? |  |  |
|  | What proportion of target joints leads to permanent joint damage? |  |  |
|  | What proportion of patients on average experiencing a major bleed die? |  |  |
| Surgeries | What proportion of patients with permanent joint damage would undergo surgery? |  |  |
|  | What proportion of surgeries is total knee replacement? |  |  |
|  | What proportion of surgeries is synovectomy? |  |  |
|  | What proportion of surgeries is radiosynovectomy? |  |  |

**RESOURCE UTILIZATION**

**Haemophilia is associated with clinical, economic and psychological burden for patients, caregivers and the wider healthcare system.**

1. **For each of the following categories, please indicate which proportion of patients visit the clinic/ hospital and provide unit costs (per visit) in local currency.**

**Please feel free to specify any other additional resources if your experience indicates differently.**

| Consultations and Hospitalization | Children (%) | | | Adult (%) | | |
| --- | --- | --- | --- | --- | --- | --- |
|  | Non-major bleed | Major bleed | Joint bleed | Non-major bleed | Major bleed | Joint bleed |
| No. of clinic visits |  |  |  |  |  |  |
| - Proportion of patients receiving clinic visits (%) |  |  |  |  |  |  |
| - Cost of clinic visit |  |  |  |  |  |  |
| No. of outpatient visits |  |  |  |  |  |  |
| - Proportion of patients receiving outpatient visits (%) |  |  |  |  |  |  |
| - Cost of outpatient visit |  |  |  |  |  |  |
| No. of inpatient visits |  |  |  |  |  |  |
| - Proportion of patients receiving inpatient visits (%) |  |  |  |  |  |  |
| - Average length of inpatient stay |  |  |  |  |  |  |
| - Cost of hospitalization (day) |  |  |  |  |  |  |

1. **For each of the following categories, please indicate in which proportion patients perform tests and scans and provide unit costs (per visit) in local currency.**

**Please feel free to specify any other additional resources if your experience indicates differently.**

| Tests and scans | Children (%) | | | Adult (%) | | |
| --- | --- | --- | --- | --- | --- | --- |
|  | Non-major bleed | Major bleed | Joint bleed | Non-major bleed | Major bleed | Joint bleed |
| No. of MRI scans |  |  |  |  |  |  |
| No. of CT scans |  |  |  |  |  |  |
| No. of ultrasounds |  |  |  |  |  |  |

1. **For each of the following categories, please indicate in which proportion of patients perform surgeries and procedures and provide unit costs (per visit) in local currency.**

**Please feel free to specify any other additional resources if your experience indicates differently.**

|  | Children | Adult |
| --- | --- | --- |
| Proportion of total knee replacement surgeries per target joint (%) |  |  |
| - Average length of hospitalization per total knee replacement (days) |  |  |
| - Length of rehabilitation per total knee replacement (days) |  |  |
| Proportion of synovectomy surgeries per target joint (%) |  |  |
| - Average length of hospitalization per synovectomy (days) |  |  |
| - Length of rehabilitation per synovectomy (days) |  |  |
| Proportion of radiosynovectomy surgeries per target joint (%) |  |  |
| - Average length of hospitalization per radiosynovectomy (days) |  |  |
| - Length of rehabilitation per radiosynovectomy (days) |  |  |

**INDIRECT COSTS**

1. **Haemophilia is associated with high indirect costs from diminished work productivity and absenteeism from work and school. Could you give us an estimate of how many days per bleed patients miss from school or work?**

| Missed days | Children (days) | Adult (days) | Caregiver (days) |
| --- | --- | --- | --- |
| No. of days of work/ school missed per NON-MAJOR BLEED |  |  |  |
| No. of days of work/ school missed per MAJOR BLEED |  |  |  |
| No. of days of work/ school missed per JOINT BLEED |  |  |  |
| No. of days of work/ school missed per non-home-based prophylaxis administration |  |  |  |

1. **Do patients with permanent joint damage receive disability benefits/ allowance?**

|  | Age range | Yes | No | If “Yes”, please provide an estimated amount of disability/ benefits allowance received. |
| --- | --- | --- | --- | --- |
| Children | 0 – 18 years old |  |  |  |
| Adults | >18 years old and above |  |  |  |

# **END**
