# Supplementary material for: Estimating the impact of improved management of haemophilia a on clinical outcomes and healthcare utilisation and costs
Source: BMC Res Notes. 2023 Nov 10;16:327. doi: 10.1186/s13104-023-06552-3 (PMC10638687; doi:10.1186/s13104-023-06552-3)
Supplement: Supplementary file 2 — Supplementary Material 2 [file 13104_2023_6552_MOESM2_ESM.docx]

## **Supplementary Files**

**Table A1. Local country HCRU and unit costs per type of bleed**

| **Bleed Treatment** | **Algeria** | | | | **Argentina** | | | | **Chile** | | | | **India** | | | |
| --- | --- | --- | --- | --- | --- | --- | --- | --- | --- | --- | --- | --- | --- | --- | --- | --- |
|  | **Non-major bleed** | **Joint bleed** | **Other major bleed** | **Unit Cost** | **Non-major bleed** | **Joint bleed** | **Other major bleed** | **Unit Cost** | **Non-major bleed** | **Joint bleed** | **Other major bleed** | **Unit Cost** | **Non-major bleed** | **Joint bleed** | **Other major bleed** | **Unit Cost** |
| **Nurse/ clinic** | 0.04 | 0.20 | 2.15 | 3 | 0.00 | 0.00 | 0.00 | 14 | 0.00 | 0.00 | 1.00 | 19 | 0.00 | 0.00 | 0.00 | 1 |
| **Outpatient** | 0.05 | 0.08 | 0.08 | 5 | 0.55 | 2.55 | 6.00 | 14 | 0.79 | 0.98 | 4.50 | 21 | 0.64 | 0.09 | 1.17 | 2 |
| **MRI** | 0.00 | 0.13 | 0.10 | 197 | 0.50 | 2.50 | 2.80 | 52 | 0.00 | 0.00 | 1.50 | 401 | 0.00 | 0.06 | 0.00 | 5 |
| **CT scan** | 0.00 | 0.00 | 1.25 | 52 | 0.38 | 0.50 | 1.38 | 41 | 0.00 | 0.00 | 2.00 | 61 | 0.00 | 0.84 | 0.50 | 4 |
| **Ultrasound** | 0.00 | 0.00 | 1.65 | 15 | 0.00 | 0.00 | 0.00 | 7 | 0.00 | 0.00 | 0.00 | 18 | 0.00 | 0.00 | 0.38 | 4 |
| **Hospitalization** | 0.00 | 0.00 | 1.00 | 5 | 0.00 | 0.00 | 1.00 | 199 | 0.50 | 1.00 | 1.00 | 11 | 0.00 | 0.09 | 0.92 | 2 |
| **Length of stay** | 0.00 | 1.30 | 11.90 | NA | 0.00 | 0.00 | 9.00 | NA | 2.50 | 3.50 | 1.00 | NA | 0.00 | 2.00 | 8.92 | NA |
| **Duration (bleed)** | 1.92 | 4.34 | 10.34 | NA | 1.92 | 2.59 | 11.83 | NA | 2.00 | 8.00 | 18.50 | NA | 1.65 | 1.84 | 6.75 | NA |
| **Orthopedic Surgery** | 6% | | | 1,708 | 3% | | | 8,714 | 27% | | | 149 | 1% | | | 2,948 |
| **Other surgeries** | 16% | | | 236 | 16% | | | 866 | 27% | | | 65 | 2% | | | 737 |
| **Rehabilitation** | 11- 26 days | | | 1 | 5 - 130 days | | | 2 | 3 - 60 days | | | 21 | 4 - 7 days | | | 1 |
| **Home Px** | 65% | | | 0 | 90% | | | 0 | 90% | | | 0 | 0% | | | 0 |
| **Clinic Px** | 35% | | | 10 | 10% | | | 14 | 10% | | | 19 | 100% | | | 1 |
| **Daily wage** | NA | | | 11 | NA | | | 17 | 0% | | | 38 | 0% | | | 3 |

**Note.** Management practices (particularly with regards likelihood of surgical intervention) are based on expert interviews and differ substantially across the different countries**.**

**Abbreviations:** HCRU: healthcare resource use; IP: Inpatient (hospitalisations); IU: international units; PWHA: patient with haemophilia A; Px: prophylaxis; Tx: treatment

| **Bleed Treatment** | **Malaysia** | | | | **Mexico** | | | | **Taiwan** | | | | **Thailand** | | | |
| --- | --- | --- | --- | --- | --- | --- | --- | --- | --- | --- | --- | --- | --- | --- | --- | --- |
|  | **Non-major bleed** | **Joint bleed** | **Other major bleed** | **Unit Cost** | **Non-major bleed** | **Non-major bleed** | **Other major bleed** | **Unit Cost** | **Non-major bleed** | **Joint bleed** | **Other major bleed** | **Unit Cost** | **Non-major bleed** | **Joint bleed** | **Other major bleed** | **Unit Cost** |
| **Nurse/ clinic** | 0.13 | 0.48 | 2.50 | 8 | 1.70 | 1.70 | 1.75 | 73 | 0.04 | 0.81 | 2.63 | 10 | 0.10 | 0.17 | 0.00 | 145 |
| **Outpatient** | 0.06 | 0.18 | 2.50 | 12 | 0.50 | 0.92 | 1.60 | 73 | 0.04 | 0.63 | 2.50 | 14 | 0.03 | 0.10 | 0.04 | 209 |
| **MRI** | 0.00 | 0.50 | 1.50 | 194 | 0.00 | 0.00 | 1.00 | 226 | 0.00 | 0.19 | 0.19 | 413 | 0.03 | 0.08 | 0.11 | 257 |
| **CT scan** | 0.00 | 0.50 | 1.50 | 109 | 0.00 | 0.45 | 2.00 | 140 | 0.00 | 0.13 | 0.13 | 181 | 0.04 | 0.05 | 0.19 | 193 |
| **Ultrasound** | 0.00 | 0.95 | 1.75 | 13 | 0.30 | 0.60 | 0.00 | 29 | 0.32 | 0.82 | 0.25 | 32 | 0.00 | 0.00 | 0.00 | 96 |
| **Hospitalization** | 0.00 | 0.25 | 0.88 | 83 | 0.33 | 0.50 | 1.00 | 466 | 0.00 | 0.13 | 0.75 | 1,492 | 0.01 | 0.09 | 0.20 | 1,333 |
| **Length of stay** | 0.00 | 1.94 | 7.38 | 0 | 1.00 | 1.50 | 13.50 | 0 | 0.00 | 1.63 | 5.83 | 0 | 2.00 | 3.00 | 7.00 | 0 |
| **Duration (bleed)** | 0.38 | 3.00 | 10.19 | 0 | 1.50 | 3.50 | 14.50 | 0 | 0.54 | 1.33 | 4.86 | 0 | 2.00 | 6.00 | 8.00 | 0 |
| **Orthopedic Surgery** | 45% | | | 5,436 | 9% | | | 5,003 | 9% | | | 705 | 1% | | | 4,819 |
| **Other surgeries** | 30% | | | 5,436 | 0% | | | 0 | 1% | | | 254 | 1% | | | 3,213 |
| **Rehabilitation** | 7 - 82 days | | | 29 | 3 - 15 days | | | 58 | 0 - 26 days | | | 5 | 11 - 17 days | | | 80 |
| **Home Px** | 86% | | | 0 | 70% | | | 0 | 85% | | | 0 | 90% | | | 0 |
| **Clinic Px** | 14% | | | 8 | 30% | | | 73 | 15% | | | 10 | 10% | | | 145 |
| **Daily wage** | NA | | | 36 | 0% | | | 20 | 0% | | | 90 | 0% | | | 32 |

**Note.** Management practices (particularly with regards likelihood of surgical intervention) are based on expert interviews and differ substantially across the different countries**.**

**Abbreviations:** HCRU: healthcare resource use; IP: Inpatient (hospitalisations); IU: international units; PWHA: patient with haemophilia A; Px: prophylaxis; Tx: treatment

**Table A2. Estimation of ABR**

| **Management** | **Patient with moderate HA** | | **Patient with severe HA** | |
| --- | --- | --- | --- | --- |
|  | Relative ABR | Calculation and source^2^ | Relative ABR | Calculation and source^2^ |
| ***OD^†^*** | *1.0* | *OD ABR differs by country and is used as benchmark ABR in calculation of Px ABR* | *1.0* | *OD ABR differs by country and is used as benchmark ABR in calculation of Px ABR* |
| **HD SHL Px^‡^** | 0.26 | Khair et al [7] report 3-yr ABR of 13.73 for OD and 3.53 for SD/HD (3.52/13.73=0.26) | 0.22 | Khair et al [7] report 3-year ABR of 16.07 for OD and 3.50 for SD (3.50/16.07=0.22) |
| **VLD SHL Px^‡^** | 0.38 | Brekkan et al [10] report relative median ABR of 1.47 with 5IU/kg 2W (VLD) versus HD (0.26*1.47=0.38) | 0.32 | Brekkan et al [10] report relative median ABR of 1.47 with 5IU/kg 2W (VLD) versus HD (0.22*1.47=0.32) |
| **LD SHL Px^‡^** | 0.34 | Brekkan et al [10] report relative median ABR of 1.34 with LD versus HD Px (0.26*1.34=0.34) | 0.28 | Brekkan et al [10] report relative median ABR of 1.34 with LD versus HD Px (0.22*1.34=0.28) |
| **ID SHL Px^‡^** | 0.27 | Brekkan et al [10] report relative median ABR of 1.04 with ID versus HD Px (0.26*1.04=0.27) | 0.23 | Brekkan et al [10] report relative median ABR of 1.04 with ID versus HD Px (0.22*1.04) |
| **Pers. SHL Px** | 0.26 | In absence of reliable data, pers HD SHL is set to SD SHL | 0.22 | In absence of reliable data, pers HD SHL is set to SD SHL |
| **EHL Px** | 0.07 | Aledort et al [8]report age-weighted ABR of 5.77 with SHL versus 1.64 with EHL (ABRs calculated from reported values, 0.26 *(1.64/5.77) =0.07) | 0.06 | Aledort et al [8]report age-weighted ABR of 5.77 with SHL versus 1.64 with EHL (ABRs calculated from reported values, 0.22 *(1.64/5.77) =0.06) |

**Notes:** *^†^* In each country, experts provided an estimate of ABR for patients managed on demand; the expected bleed rate for alternate regimens was estimated based on published literature common across all analyses. The OD patients are taken as the benchmark in order to factor in country differences in underlying bleed rates; **^‡^**Local searches highlighted wide disparity in ABR estimates for lower dose regimens; generalised data from the Brekkan et al simulations [10] were used in preference; ^§^The relative estimates for ABR are simplified calculations based on available data from disparate populations and should be re-visited as and when better comparative data become available

**Abbreviations:** 2W: twice weekly dosing regimen; 3W three times weekly dosing regimen; ABR: annualised bleed rate; EHL: extended half-life; HD: high- dose; ID: intermediate-dose; LD: low-dose; Pers.: personalised; Px: prophylaxis; SHL: standard-half life; VLD: very low-dose
